# Renal Function Interferes with Copeptin in Prediction of Major Adverse Cardiac Events in Patients Undergoing Vascular Surgery

**DOI:** 10.1371/journal.pone.0123093

**Published:** 2015-04-13

**Authors:** Claudia Schrimpf, Hans-Joerg Gillmann, Bianca Sahlmann, Antje Meinders, Jan Larmann, Mathias Wilhelmi, Thomas Aper, Saad Rustum, Ralf Lichtinghagen, Gregor Theilmeier, Omke E. Teebken

**Affiliations:** 1 Division of Vascular & Endovascular Surgery, Department of Cardiothoracic, Transplantation and Vascular Surgery, Hannover Medical School, Hannover, Germany; 2 Department for Anesthesiology and Intensive Care Medicine, Hannover Medical School, Hannover, Germany; 3 Department for Clinical Chemistry, Hannover Medical School, Hannover, Germany; The University of Tokyo, JAPAN

## Abstract

**Objective:**

Precise perioperative risk stratification is important in vascular surgery patients who are at high risk for major adverse cardiovascular events (MACE) peri- and postoperatively. In clinical practice, the patient’s perioperative risk is predicted by various indicators, e.g. revised cardiac index (RCRI) or modifications thereof. Patients suffering from chronic kidney disease (CKD) are stratified into a higher risk category. We hypothesized that Copeptin as a novel biomarker for hemodynamic stress could help to improve the prediction of perioperative cardiovascular events in patients undergoing vascular surgery including patients with chronic kidney disease.

**Methods:**

477 consecutive patients undergoing abdominal aortic, peripheral arterial or carotid surgery from June 2007 to October 2012 were prospectively enrolled. Primary endpoint was 30-day postoperative major adverse cardiovascular events (MACE).

**Results:**

41 patients reached the primary endpoint, including 63.4% aortic, 26.8% carotid, and 9.8% peripheral surgeries. Linear regression analysis showed that RCRI (P< .001), pre- (P< .001), postoperative Copeptin (P< .001) and Copeptin level change (P= .001) were associated with perioperative MACE, but CKD remained independently associated with MACE and Copeptin levels. Multivariate regression showed that increased Copeptin levels added risk predictive information to the RCRI (P= .003). Especially in the intermediate RCRI categories was Copeptin significantly associated with the occurrence of MACE. (P< .05 Kruskal Wallis test). Subdivision of the study cohort into CKD stages revealed that preoperative Copeptin was significantly associated with CKD stages (P< .0001) and preoperative Copeptin measurements could not predict MACE in patients with more severe CKD stages.

**Conclusion:**

Preoperative Copeptin loses its risk predictive potential for perioperative MACE in patients with chronic kidney disease undergoing vascular surgery.

## Introduction

Patients undergoing vascular surgery are prone to perioperative cardiovascular events and progressive organ dysfunction due to an often generalized vascular pathology. Therefore, clinicians aim for precise risk prediction to guide therapeutic management preoperatively, but current risk prediction strategies lack sufficient accuracy. Mostly, the patients' risk is stratified by using clinical risk scores such as the Revised Cardiac Risk Index (RCRI)[1] and derivations thereof [2,3]. In clinical routine these scores are easy to use and they help the physician decide on peri- and postoperative therapy. Generally, these risk scores are useful to compare cohort event rates, but unfortunately they do not allow sufficient individual risk estimation. This is partly due to the fact that vascular surgery patients accumulate cardiovascular risk factors and many have already experienced cardiovascular events, which will place most of these patients homogenously in higher risk categories of clinical scores [1] making a distinct decision on resource allocation difficult or impossible for the physician.

Biomarkers may improve risk predictive models in the future. For example, has Copeptin recently been demonstrated to be suitable for guiding management of patients with acute chest pain [4,5]. Within the last few years, a role for Copeptin as a risk predictive biomarker in the management of acute myocardial infarction, chronic heart failure, stroke as well as chronic kidney disease (CKD) has been reported, but perioperative data are sparse [6,7,8,9]. A small study using a mixed primary endpoint of early and late adverse outcomes has identified Copeptin as a biomarker for long term survival in vascular surgery patients without impaired kidney function, but failed to show unequivocal association with immediate outcome within 30 days after surgery although the data were suggestive [10].

Copeptin is liberated from preprovasopressin as the 39-amino acid glycosylated carboxyl-terminal part and released in isostoichiometric amounts to arginine-vasopressin (AVP) [11,12]. AVP is an effective osmoregulator that can increase peripheral vasoconstrictive activity through interaction with its receptor V1 [11,12]. On the other hand, binding to the V2 receptor mediates water retention in renal tubules [13]. Unfortunately, the circulatory half-life of AVP is very short rendering it inaccessible for clinical routine determination. In contrast to AVP, Copeptin is a highly stable protein easily quantifiable in patients’ plasma and serum. Due to its close correlation to AVP it can therefore be used to estimate AVP. Since Copeptin can thus be viewed as a surrogate marker of hemodynamic stress, it may improve perioperative risk prediction [14].

We hypothesized that Copeptin (as a marker of hemodynamic stress) in combination with the RCRI (as an established risk predictive clinical score) may deliver differential risk predictive value and lead to improved risk stratification for vascular surgery patients with respect to the prediction of perioperative major adverse cardiac events (MACE).

## Material and Methods

### Study design and population

The study was approved by the ethics committee of Hannover Medical School (approval no. 4598). In total, 727 patients underwent elective aortic, peripheral artery, or carotid artery surgery with an overall mortality of below 1.5% from 6/2007 until 10/2012. Of these, 477 consecutive patients gave written informed consent and were prospectively enrolled in the study. Emergency patients, patients with endovascular treatment and patients under the age of 18 years were excluded. Due to personnel reasons blood and tissue collection and processing was not possible for several weeks. During this time no patients were recruited. Clinical parameters (height, weight, sex, age), RCRI items [1] and medical history (history of stroke, arterial hypertension, chronic obstructive pulmonary disease (COPD), diabetes mellitus, coronary artery disease (CAD), chronic kidney disease (CKD assessed by calculated glomerular filtration rate (GFR)), heart failure were collected from medical records.

### Study endpoint

The primary endpoint was the 30-day risk of MACE, defined as the occurrence of (1) spontaneous myocardial infarction (MI type I) or a myocardial infarction secondary to an ischemic imbalance (MI type II), (2) cardiovascular death (MI type III), each according to current guideline criteria [15] or (3) any new rise of cardiac troponin measurements prompted by clinical suspicion for an acute coronary syndrome (cut-off: >50 ng/L for the 5^th^ generation hs-cTnT assay, 0.05 μg/L for the 3^rd^ generation cTnT assay;) [15,16] until 30 days after surgery. To complete the 30-day time interval, data regarding MI and consecutive treatment were collected by standardized phone interviews.

### Biochemical biomarker and clinical score assessment

For each patient, blood samples were drawn immediately before surgery (preoperative sample) and on postoperative day one (postoperative sample). Peri- as well as postoperative complications within a 30-day period were recorded.

Blood samples were stored at -80°C until assayed in one batch after the end of data collection. Copeptin was measured using the BRAHMS Kryptor Assay (Thermo Fisher scientific, Waltham, MA, USA) with a lower detection limit of 4.8 pmol/L (data from manufacturer), resulting in a minimum for Copeptin of ≤ 4.8 pmol/L.

The RCRI was calculated according to Lee et al., where defined pre-existing diseases (history of cerebrovascular disease, ischemic heart disease, congestive heart failure, diabetes or renal failure) and surgical procedures with identified high perioperative risk each score one point [1]. For analysis RCRI groups were defined as follows: RCRI 0: no point given according to Lee et al, RCRI 1: one point, RCRI 2: two points and RCRI ≥3: more than three points calculated. RCRI 2 was defined as intermediate, RCRI ≥3 as high-risk category.

### Statistical analysis

Data were compared using nonparametric tests as suited, Mann-Whitney U for group wise comparisons, Kruskal-Wallis H followed by Dunn’s test for comparing multiple groups.

The association of Copeptin (pre-, postoperatively and perioperative changes) with pre-existing disease entities and patients’ demographical data as well as clinical score (RCRI) and type of surgery was analyzed by linear regression analysis. Prognostic factors showing significance in univariate regression (P<.1) were applied to further multivariate analysis.

As high-sensitive-cardiac Troponin-T (hs-cTnT) is a clinically used marker for myocardial infarction postoperative Copeptin was correlated to postoperative hs-cTnT. MACE association with pre-existing disease entities, patients’ demographical data, clinical score, Copeptin level and surgical procedure was assessed accordingly. Results are presented as median and range.

Further analysis was performed for type of surgery (i.e. aortic, peripheral, or carotid artery surgery; Table 1). Again, regression analysis was conducted to examine Copeptin and MACE prediction according to surgery type.

**Table 1:** Demographical data of study population subdivided into surgical procedures.

Variable	Total	Aorta	Peripheral	Carotid	P-value
Number of patients n (%)	477 (100)	189 (39.6)	98 (20.5)	190 (39.8)	
Age (years) median (25–75 percentile)	70 (63–75)	69 (60–74)	70 (63–76)	71 (65–76)	.043
male sex n (%)	382 (79.9)	171 (44.8)	79 (20.7)	132 (34.6)	.002
Weight (kg) median (25–75 percentile)	80 (70–90)	82 (73.0–92.5)	80 (71–89)	75.5 (69–75)	.01
History of Stroke n (%)	102 (21.4)	17 (16.7)	14 (13.7)	71 (69.6)	<.001
CAD n (%)	181 (37.8)	71 (39.2)	35 (19.3)	75(41.4)	.52
RCRI median		2	2	1	<.001
RCRI 0 n (%)	51 (10.7)	1 (0.5)	2 (2)	48 (25.3)	
RCRI 1 n (%)	193 (40.5)	75 (39.7)	38 (38.8)	80 (42.1)	
RCRI 2 n (%)	135 (28.3)	66 (34.9)	26 (26.5)	43 (22.6)	
RCRI ≥3 n (%)	98 (20.5)	47 (24.9)	32 (32.7)	19 (10%)	
GFR median (25–75 percentile) (mL/min/1.73m^2^)	60 (55–60)	60 (55.5–60)	60 (51.8–60)	60 (56–60)	.05
Copeptin preop (pmol/L) median (25–75 percentile)	10.16 (5.67–18.07)	10.99 (6.6–19.5)	10.73 (6.03–19.1)	8.64 (≤4.8–16.3)	.08
Copeptin postop (pmol/L) median (25–75 percentile)	23.55 (11.50–59.9)	46.03 (20.0–97.5)	20.62 (10.08–42.3)	15.37 (8.8–28.7)	.03
Copeptin delta absolute (pmol/L) median (25–75 percentile)	12.88 (4.01–44.88)	38.47 (11.1–85.1)	8.96 (2.1–29.1)	7.91 (2.2–17.5)	.16
MACE n (%)	41 (8.6)	26 (5.5)	4 (0.8)	11 (2.3)	.004

P depicts P value of univariate linear regression calculated for each variable and type of surgery. The number of patients (n) for each group as well as percentage (%) is depicted. Other variables are shown as median with 25–75 percentile. Abbreviations are used as follows: coronary artery disease (CAD), revised cardiac risk index (RCRI), glomerular filtration rate (GFR), preoperative values for Copeptin (preop Copeptin), postoperative values for Copeptin (postop Copeptin), change of Copeptin levels between pre- and postoperative sample (Copeptin delta absolute), Major adverse cardiovascular events (MACE).

Another subgroup analysis was performed subdividing the study population into patients with or without CKD, categorizing groups according to National Kidney Foundation guidelines [17]. These were analyzed for the occurrence of MACE in each subgroup according to preoperative Copeptin measurements. Groups were analyzed by Mann-Whitney U test.

Hypothesis-testing was two-tailed, a p-value <.05 was considered significant. Statistical analysis was performed with SPSS 20.0 (SPSS Inc., Chicago, IL) GraphPad Prism 4 (San Diego, California USA) and MedCalc 12.2.1.0 (MedCalc Software, Ostende, Belgium).

## Results

### Patient characteristics

Demographics from 477 patients are summarized in Table 1. Forty-one patients (8.6%) reached the primary endpoint MACE (Table 1). Postoperative 30-day mortality was 0.2% (1/477 patients). On postoperative day zero 4 individuals (9.8%) suffered MACE, followed by 17 individuals (41.5%) on day one, five cases (12.2%) on day 2, eight (19.5%) on day 3 and 7 patients (17.1%) on day 4 or on later time points. Median preoperative Copeptin level was 10.16 (5.67–18.07; 25–75 percentile) pmol/L; and increased significantly (P<.001) to a postoperative Copeptin median of 23.55 (11.50–59.9; 25–75 percentile) pmol/L with a respective median Copeptin change of 12.88 (4.01–44.88; 25–75 percentile) pmol/L.

### Clinical risk factors and MACE

Univariate analysis demonstrated an association of MACE with pre-existing comorbidities i.e. heart failure, COPD, CAD, gender and CKD (Table 2). In the consecutive multivariate analysis CKD or a history of heart failure remained significantly associated with MACE.

**Table 2:** Linear regression analysis of MACE and existing comorbidities.

Epidemiology and comorbidities	Significance univariate	Significance multivariate
Sex	.089*	.111
Age	.680	
Weight	.850	
Height	.911	
BMI	.892	
Hypertension	.974	
Diabetes	.216	
History of stroke	.375	
Preoperative GFR (mL/min/1.73m^2^)	<.001*	<.001*
Smoking	.282	
Heart failure	<.001*	.002*
COPD	.034*	.247
CAD	.030*	.445
RCRI category	<.001*	<.001*
Surgical procedure	.844	
Copeptin preop	<.001*	.003*
Copeptin postop	<.001*	.234
Copeptin change	.001*	.027

*marks significant confounders.

Abbreviations are used as follows: BMI (Body mass index), TIA (transitory ischemic attack) COPD (chronic obstructive pulmonary disease), CAD (coronary artery disease), RCRI (Revised cardiac index), Surgical procedure (type of surgery performed subdivided into aortic, carotid and peripheral arterial procedures). Copeptin preoperative, postoperative and Copeptin change; significant results were additionally used in multivariate regression (significance multivariate) results are shown with p values. Comorbidities and putative risk predictors were analyzed in separate univariate and multivariate analysis.

### Clinical Risk factors and Copeptin

Results of linear regression followed by a multivariate regression are reported in Table 3. Preoperative CKD, height, gender, as well as history of CAD, hypertension, RCRI category and type of surgery were associated with elevated preoperative Copeptin levels (Table 3). Postoperative Copeptin was associated with CKD, gender, history of stroke, CAD and hypertension as well as RCRI and surgical procedure. Changes of Copeptin within the perioperative time course were related to CKD, history of stroke, CAD, RCRI category and surgical procedure (Table 3). Multivariate analysis again revealed pre-existing CKD as well as RCRI category and history of stroke influencing Copeptin levels (Table 3).

**Table 3:** Analysis of Copeptin levels.

	Preoperative Copeptin		Postoperative Copeptin		Copeptin change	
Epidemiology and comorbidities	univariate	multivariate	univariate	multivariate	univariate	multivariate
Sex	.009*	.025*	.096*	.108	.451	
Age	.249		.358		.585	
Weight	.179		.788		.773	
Height	.081*	.690	.427		.980	
BMI	.530		.976		.807	
Hypertension	.028*	.506	.072*	.776	.277	
Diabetes	.484		.470		.603	
History of stroke	.678		.014*	.001*	.008*	<.001*
Preoperative GFR (mL/min/1.73m^2^)	<.001*	<.001*	<.001*	<.001*	<.001*	<.001*
Smoking	.722		.980		.851	
Heart failure	.910		.582		.494	
COPD	.168		.349		.643	
CAD	.002*	.317	.003*	.407	.048*	.318
RCRI category	<.001*	.942	<.001*	.020*	<.001*	.006*
Surgical procedure	.077*	.745	.033*	.377	.095*	.146

Linear regression analysis of pre-, postoperative Copeptin levels and Copeptin change (pmol/L) and existing comorbidities as well as RCRI category and surgical procedure,

*marks significant confounders, abbreviations are used as follows: BMI (Body mass index), TIA (transitory ischemic attack), COPD (chronic obstructive pulmonary disease), CAD (coronary artery disease), RCRI (Revised cardiac index), surgical procedure (type of surgery performed subdivided into aortic, carotid and peripheral surgeries). Significance reveals results of linear regression analysis; significant results were additionally used in multivariate regression (significance multivariate) results are shown with P values.

### Copeptin and MACE

**Fig 1:**
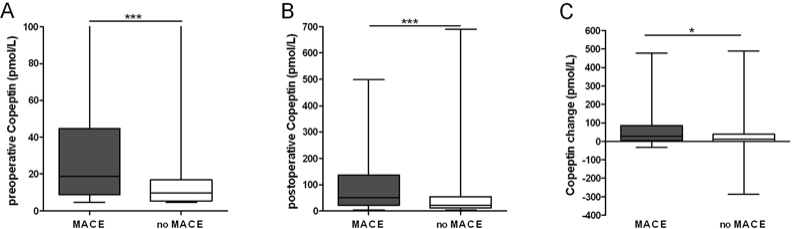
Copeptin is elevated in patients sustaining Major Adverse Cardiovascular Events (MACE) throughout the perioperative phase. Boxplots of pre- (A) and postoperative (B) Copeptin levels as well as perioperative Copeptin change (C) (pmol/L). Groups were analyzed by Mann-Whitney U test (A) P = .0001, (B) P = .0002, (C) P = .014.

Patients reaching the endpoint MACE had significantly elevated median preoperative Copeptin levels compared to patients not reaching the endpoint 18.89 pmol/L (≤4.80 pmol/L—180.7 pmol/L) versus 9.75 pmol/L (≤4.80 pmol/L—321.6 pmol/L, P<.001; Fig 1).

### Association of Copeptin and postoperative high sensitive Troponin T

Postoperative hs-cTnT levels ranged from <3.0 to 1499.0 ng/L (median 13.8ng/L). The correlation between postoperative hs-cTnT and postoperative Copeptin was weak and likely not clinically relevant (r = .170, P = .001).

### MACE prediction using RCRI and Copeptin

**Fig 2:**
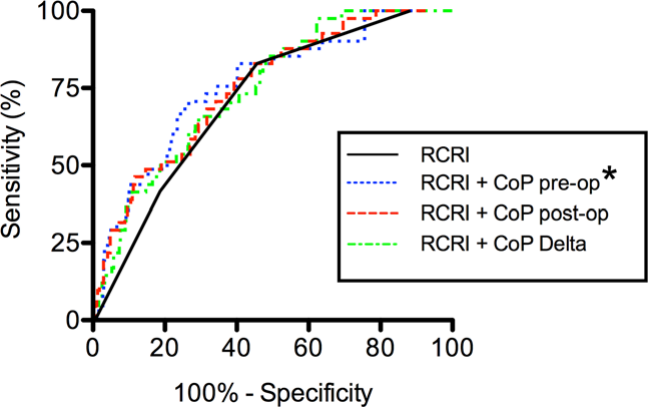
ROC analysis comparing the RCRI alone or combined with Copeptin-derived parameters. Only preoperative Copeptin (blue dotted line) improved risk predictive accuracy of the RCRI (P = .0371, AUC .752). The RCRI-ROC Curve (black line) (AUC .714) indicates prediction of the occurrence of major adverse cardiovascular events (MACE). The combination of RCRI and postoperative Copeptin (red dashed line) (P = .0620, AUC .751) and RCRI and Copeptin changes (P = .1525, AUC .710) during the perioperative course (green dashed and dotted line) do not reach significantly larger AUCs. * marks significant values.

The capacity to predict MACE by RCRI category, Copeptin pre- / postoperative values or change was tested in linear and multivariate regression models (Table 2). Surgical procedure was not significantly associated with MACE (P = .844). However, RCRI (P<.001), preoperative Copeptin (P<.001), postoperative Copeptin (P<.001) and Copeptin change (P = .001) were associated with MACE. Multivariate analysis revealed RCRI (P<.001) and preoperative Copeptin (P<.003) to be significantly associated with MACE. The DeLong method was used to analyze the improvement in risk predictive accuracy of RCRI and RCRI in combination with pre- and postoperative Copeptin levels as well as Copeptin change. Only preoperative Copeptin levels were able to improve ROC curves significantly compared to RCRI alone (P = .037; Fig 2). For patients in the intermediate or high RCRI groups the AUC was larger when preoperative Copeptin level was added to the RCRI (Fig 2). When each RCRI category was subdivided into patients with and without MACE, preoperative Copeptin levels were significantly higher in MACE patients in the intermediate RCRI group (RCRI 2, P<.05; Table 4).

**Table 4:** Preoperative Copeptin levels (pmol/L) in patients with or without MACE according to RCRI level.

RCRI	MACE +	MACE -	MACE +/-
**RCRI 0 (n)**	0	(51)	(51)
(min-max) median	(n.a.) n.a.	(≤4.8–78.01) 6.66	(≤4.8–78.01) 6.66
**RCRI 1 (n)**	(7)	(186)	(193)
(min-max) median	(≤4.8–23.31) 4.8	(≤4.8–163.8) 9.56	(4.8–163.8) 9.35
**RCRI 2 (n)**	(17)	(118)	(135)
(min-max) median	(≤4.8–180.7) 19.18	(≤4.8–274.5) 9.77	(≤4.8–274.5) 10.22
**RCRI ≥ 3 (n)**	(17)	(81)	(98)
(min-max) median	(6.14–165.7) 31.79	(≤4.8–321.6) 15.78	(≤4.8–321.6) 17.29
**All (n)**	(41)	(436)	(477)
(min-max) median	(≤4.8–180.7) 18.89	(≤4.8–321.6) 9.75	(≤4.8–321.6) 10.16

### Copeptin predicting MACE in CKD and non-CKD patients

**Fig 3:**
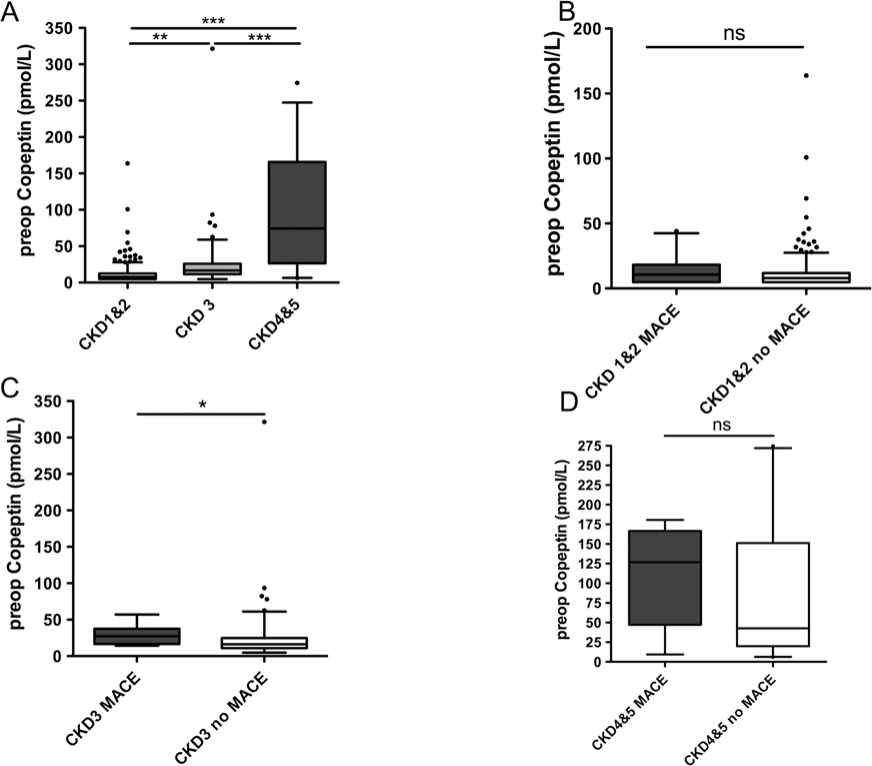
Copeptin interferes with kidney injury in prediction of MACE. Preoperative Copeptin levels (pmol/L) are significantly (P<.0001) elevated in patients with chronic kidney disease increasing with severity of kidney injury (A). Preoperative Copeptin is not associated with MACE in patients with CKD 1&2 (B) (P = .3787) or CKD 4&5 (D) (P = .2264) but shows significant association with MACE in CKD 3 (C) (P = .0163). Data were analyzed using Mann Whitney U test for comparing two groups and Kruskal Wallis test followed by Dunns test for multiple comparisons. Blots are depicted as 5–95 percentile.

As Copeptin was associated with MACE (Table 2) as well as declining kidney function (Table 3) in univariate analysis, the interdependence of these parameters was analyzed (Fig 3). Only preoperative Copeptin, but not postoperative measurements or perioperative change was independently associated with MACE in multivariate analysis (Table 2). Individuals reaching the endpoint MACE in the whole study cohort showed a median preoperative Copeptin of 18.89 pmol/L (≤4.80 pmol/L to 180.70 pmol/L), while patients not reaching the endpoint had a preoperative Copeptin of 9.75 pmol/L (80 pmol/L to 321.60 pmol/L). CKD itself was associated with a significant preoperative Copeptin increase (Fig 3a, P<.0001). Therefore the study cohort was categorised into CKD stages and MACE only in relation to preoperative Copeptin. Preoperative Copeptin in patients with CKD stage 1&2 (GFR ≥ 60 mL/min/1.73m^2^; n = 335) was 7.97 pmol/L (median) (≤4.80 pmol/L to 163.80 pmol/L). Patients with CKD stage 3 (GFR 59–30 mL/min/1.73m^2^;n = 112) had a preoperative Copeptin of 16.62 pmol/L (≤4.80 pmol/L to 321.60 pmol/L). Copeptin in patients with CKD stage 4&5 (GFR ≤ 29 mL/min/1.73m^2^ and patients on dialysis n = 30) was 74.39 pmol/L in median (6.31 pmol/L to 274.5 pmol/L). Each CKD stage was subdivided into patients reaching and not reaching the primary endpoint MACE (Fig 3). In Stage 1&2 20 of 335 patients, in CKD 3 10 patients of 112 and in stage 4&5 10 out of 30 patients suffered MACE.

## Discussion

This prospective study demonstrates the limitation of Copeptin in MACE prediction in patients with impaired kidney function who undergo vascular surgery.

Although Copeptin in combination with RCRI improved risk predictive accuracy for MACE in all vascular surgery patients including those with CKD, the association for Copeptin increase with a reduced glomerular filtration rate makes a distinction in Copeptin elevation due to a cardiovascular event or due to impaired kidney function difficult. (Fig 3). Although Copeptin improves risk stratification in combination to the RCRI, its usefulness in the subpopulation of patients with impaired kidney function should prompt caution. Preoperative Copeptin levels in this subpopulation of patients showed huge variability, ranging from a minimum of ≤4.80 pmol/L to a maximum of 321.60 pmol/L. To date, preoperative identification of patients that will suffer a perioperative myocardial infarction lacks predictive accuracy. This study aimed to help improving perioperative cardiovascular risk prediction via Copeptin in patients undergoing vascular surgery. Like hs-cTnT and RCRI, Copeptin levels are associated with the occurrence of MACE (Fig 1) [16]. Our data revealed a rather weak correlation of postoperative Copeptin to hs-cTnT levels, pointing at the differential information that these biomarkers are thought to add, i.e. increased Copeptin levels indicate hemodynamic stress while Troponin T is interpreted as a marker of myocardial injury [16,18].

Copeptin levels have been shown to be associated with cardiovascular disease in patients suffering from end stage renal disease [19,20]. In a previous perioperative study, which excluded patients with renal disease, Copeptin levels were significantly associated with creatinine clearance and GFR [10]. In our study, increased severity of CKD was associated with increased levels of Copeptin (Fig 3), verifying recent studies, which linked elevated Copeptin levels to decreased kidney function [20] and showed elevated Copeptin levels in patients with type II Diabetes mellitus [19].

In this study, we were able to show, that Copeptin may help in the prediction of MACE in addition to the RCRI (Fig 2) in vascular surgery patients, but lacks good predictive accuracy in patients that suffer from CKD (Fig 3).

Copeptin has proven superior to NTproBNP in predicting increased short and long-term cardiovascular risk in vascular surgery patients without chronic kidney disease [10]. NTproBNP, as a marker of myocardial strain, and Copeptin as marker of cardiovascular stress both relate to myocardial dysfunction. In their study evaluating the additive value of perioperative Copeptin, Jarai et al. excluded patients with an elevated creatinine of ≥1.4mg/dL, but CKD still remained an independent variable in multivariate linear regression analysis. Because an elevated creatinine in their study accounted only for 18% of the increased Copeptin levels, this finding was not examined in more detail and left unattended. Although we demonstrate that a preoperative Copeptin measurement is able to predict MACE (Fig 1) in all vascular surgery patients, we were eager to more specifically address the potential role of Copeptin for risk prediction in patients suffering from CKD. CKD was an independent contributor in multivariate analysis to the prediction of MACE (Table 2) and Copeptin levels (Table 3). Subgroup analysis revealed that Copeptin in patients suffering from CKD was not useful to predict MACE (Fig 3), but its level increases with declining kidney function. Therefore our data adds valuable information to Jarai's study as we can demonstrate that Copeptin in this specialised subpopulation of patients is not useful to predict MACE.

Maravic-Stojkovic et al. [21] showed that Copeptin levels were elevated in patients suffering from a perioperative stroke after carotid endarterectomy. In our study out of 190 patients presenting for carotid endarterectomy, 71 with a history of stroke were included (37,7%). We were neither able to demonstrate a significant difference in preoperative Copeptin levels in patients with or without a history of stroke nor in symptomatic vs. asymptomatic carotid endarterectomy patients (S1 Fig). The interpretability of our data is limited because the rate of peri- and postoperative stroke events in carotid endarterectomy patients was rather low (n = 8 (4.2%), including 6 patients with former history of stroke (3.15%) and only 2 patients with a perioperative stroke (1.05%)).

Preoperative risk assessment should be accurate, quick and easy to use as well as available for every patient [22]. We propose that preoperative Copeptin in combination with clinical score assessment can be used to identify patients at risk (Fig 2) but not in patients with CKD. Instead, CKD should be considered as one of the key independent factors in MACE prediction.

Additional biomarker testing in itself increases costs. Therefore it is necessary to exclude biomarkers that are not suitable to improve risk prediction to allow development of targeted resource allocation. Copeptin can be considered for MACE prediction in vascular surgery patients with limited usefulness in patients with impaired kidney function.

### Study limitations

If we had actively searched for patients reaching the primary endpoint MACE via 72h-Holter ECG, 12-lead ECGs or serial troponin measurements, we would likely have found more MACE cases [23]. Therefore, it might be possible that we did not detect clinically in apparent cases and underestimated the power of Copeptin measurements.

Furthermore, our findings on CKD patients rely on post-hoc subgroup analysis. The group of patients with CKD 1&2 was rather large with 335 patients, but CKD 4&5 only consisted of 30 patients in total, of who 10 had MACE. A larger study in patients suffering from CKD that could now be planned based on our subgroup analysis is necessary to unequivocally rule out the usefulness of Copeptin for the prediction of MACE in this population.

## Supporting Information

S1 Fig Copeptin in symptomatic vs. asymptomatic CEAs. Preoperative Copeptin levels show no significant difference in symptomatic vs. asymptomatic (P = 0.067) and stroke vs. no stroke (P = 0.455) CEA patients using Mann Whitney U test.
(JPG)

S1 Table Data table. Underlying Data table.
(XLSX)
