# Supplementary figures and images for: Renal Function Interferes with Copeptin in Prediction of Major Adverse Cardiac Events in Patients Undergoing Vascular Surgery
Source: PLoS One. 2015 Apr 13;10(4):e0123093. doi: 10.1371/journal.pone.0123093 (PMC4395325; doi:10.1371/journal.pone.0123093)

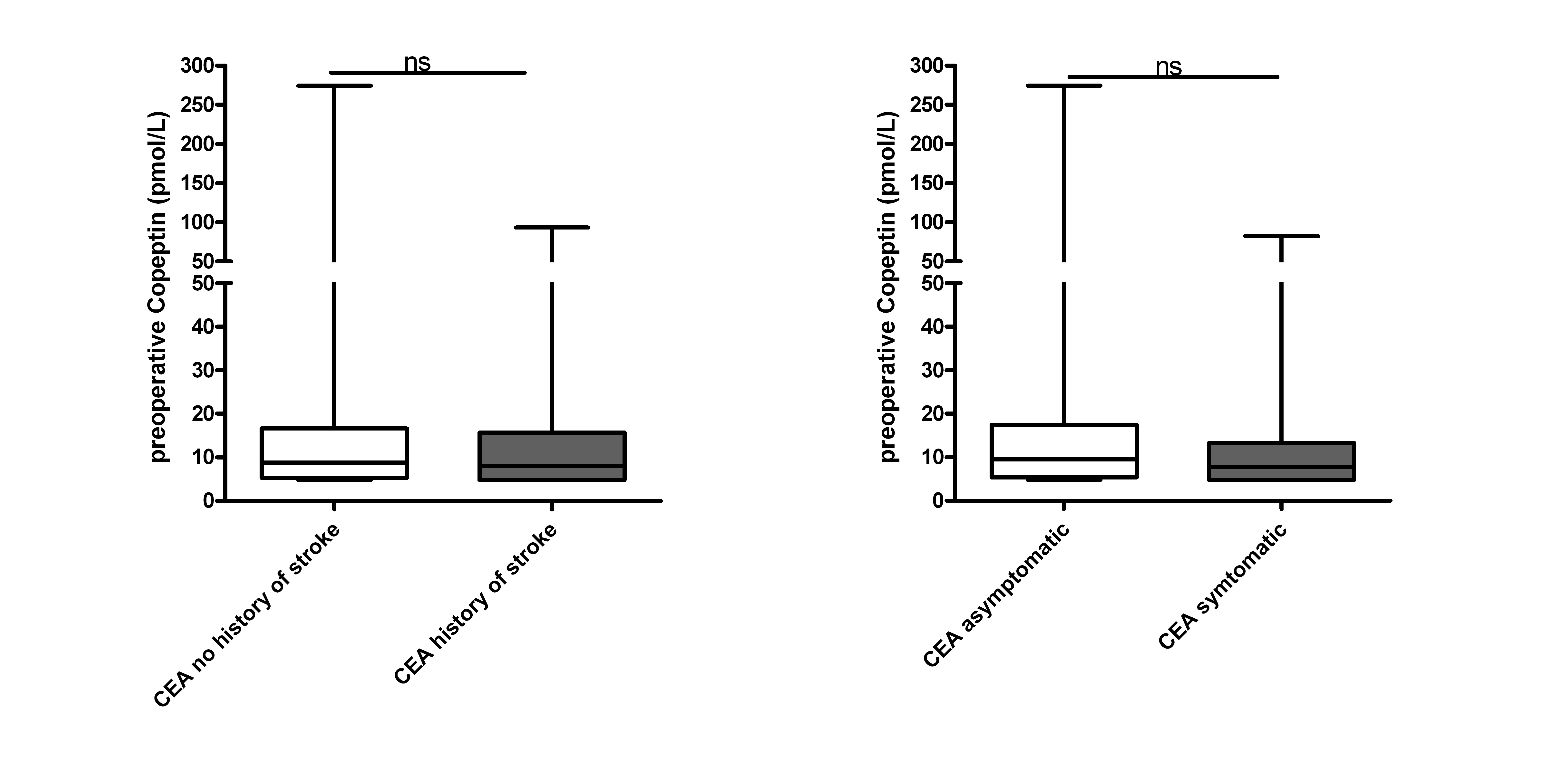

Supplement: S1 Fig — Preoperative Copeptin levels show no significant difference in symptomatic vs. asymptomatic (P = 0.067) and stroke vs. no stroke (P = 0.455) CEA patients using Mann Whitney U test. (JPG) [file pone.0123093.s001.jpg]
